# Coexistence of GLIM-defined malnutrition and sarcopenia have negative effect on the clinical outcomes in the elderly gastric cancer patients after radical gastrectomy

**DOI:** 10.3389/fnut.2022.960670

**Published:** 2022-08-19

**Authors:** Wei-Zhe Chen, Xian-Zhong Zhang, Feng-Min Zhang, Ding-Ye Yu, Wen-Hao Chen, Feng Lin, Qian-Tong Dong, Cheng-Le Zhuang, Zhen Yu

**Affiliations:** ^1^Department of Gastrointestinal Surgery, Shanghai Tenth People's Hospital, Tongji University School of Medicine, Shanghai, China; ^2^Department of General Surgery, Shanghai Ruijin Hospital, Shanghai Jiao Tong University School of Medicine, Shanghai, China; ^3^Department of Gastrointestinal Surgery, The First Affiliated Hospital of Wenzhou Medical University, Wenzhou, China

**Keywords:** elderly patients, gastric cancer, GLIM, malnutrition, sarcopenia

## Abstract

**Background:**

Malnutrition and sarcopenia are common in elderly gastric cancer patients, which are also interrelated and affect each other. We aimed to determine the characteristics of coexistence of malnutrition and sarcopenia in the elderly gastric cancer patients and investigate the predictive roles of malnutrition and sarcopenia on clinical outcomes.

**Methods:**

Between 2014 and 2019, a total of 742 elderly gastric cancer patients were enrolled. Malnutrition and sarcopenia were diagnosed according to the most recent diagnostic criteria. Patients were divided into four groups according to presence of these two symptoms. Clinical characteristics, short- and long-term outcomes were compared among four groups. The independent risk factors for complications and survival were evaluated using univariate and multivariate analyses.

**Results:**

Of all patients, 34.8% were diagnosed with malnutrition and 34.0% were diagnosed with sarcopenia. Patients with both malnutrition and sarcopenia had the highest rate of total (*P* < 0.001), surgical (*P* = 0.003), and medical complications (*P* = 0.025), and the highest postoperative hospital stays (*P* < 0.001) and hospitalization costs (*P* < 0.001). They also had the worst overall survival (*P* < 0.0001) and disease-free survival (*P* < 0.0001). Sarcopenia and Charlson Comorbidity Index (≥2) were independent risk factors for total complications. Hypoalbuminemia and malnutrition were non-tumor-related independent risk factors for overall survival and disease-free survival.

**Conclusions:**

Malnutrition and sarcopenia had superimposed negative effects on elderly gastric cancer patients. Preoperative geriatric evaluation including screening for malnutrition and sarcopenia are recommended for all elderly gastric cancer patients for accurate treatment strategy.

## Introduction

Gastric cancer is one of the critical cancers, with more than one million new cases worldwide in 2020, ranking fifth in incidence and fourth in mortality (1). Surgery remains the primary approach for the treatment of gastric cancer. With the increase in the aging society, gastric cancer has been increasingly diagnosed in elderly patients. Studies have reported that age and aging are risk factors for postoperative mortality and overall survival (OS) after radical gastrectomy (2). As a malignant tumor of the gastrointestinal tract, gastric cancer may often cause nausea, vomiting, or anorexia in patients, resulting in inadequate dietary intake and protein synthesis, thereby leading to malnutrition. Tumor-associated malnutrition is characterized by increased protein catabolism that can lead to a negative protein balance. Furthermore, malnutrition is also an age-related disease common in elderly gastric cancer patients (3). In addition, malnutrition in elderly patients has been reported to critically impact the functional improvement during rehabilitation, postoperative complications, mortality, and rehospitalization (4–6).

The study of human components, especially sarcopenia, has been currently attracting increasing interest in the fields of geriatrics and oncology. Sarcopenia is a syndrome caused by multifactorial etiology, including tumor and age, and is characterized by a systemic loss of skeletal muscle mass as well as a decrease in muscle strength and performance (7). Elderly patients commonly suffer from a variety of comorbidities, such as sarcopenia, due to the functional decline of the body. The prevalence of sarcopenia was estimated to be as high as 50% among people aged ≥ 80 years (8), which posed burdens to the health and economics of the population. A growing number of studies have confirmed the negative impacts of sarcopenia on the short- and long-term postoperative outcomes in elderly cancer patients (9–12).

Although malnutrition and sarcopenia are two different symptoms, they are interrelated and affect each other. Malnutrition is usually associated with reduced protein intake (13), which is the basis of muscle metabolism. Nutrition and physical activity are two important factors affecting sarcopenia, and appropriate nutritional interventions may alleviate sarcopenia. A study has suggested that fully diagnosed malnutrition may accurately predict the incidence of sarcopenia (14). When malnutrition and sarcopenia occur simultaneously, it may lead to malnutrition-sarcopenia syndrome (MSS) (15), which increases the risk of mortality more than malnutrition or sarcopenia alone (16, 17). Since both are associated with age-related changes, elderly patients are particularly vulnerable to coexistence of malnutrition and sarcopenia. A recent mate-analysis also revealed that the association between and prevalence of sarcopenia and malnutrition in elderly hospitalized patients is substantial (18). However, the concept of MSS has not been widely examined, and its significance in clinical practice and research is poorly understood (19).

Elderly gastric cancer patients should be detected for malnutrition and sarcopenia using a comprehensive preoperative geriatric assessment for individualized perioperative patient management, as it facilitates the improvement of outcomes after radical gastrectomy. However, no reports on the effects of the coexistence of sarcopenia and malnutrition on elderly gastric cancer patients are available. Recently, the Global Leadership Initiative on Malnutrition (GLIM) published a global consensus on the core diagnostic criteria for adult malnutrition in different clinical settings. This diagnostic criterion is expected to be recognized by various international nutritional associations in the future. In addition, the phenotypic and etiologic criteria used to diagnose sarcopenia and cachexia should also be validated (20, 21).

The aim of this study is to report the prevalence and characteristics of coexistence of GLIM-defined malnutrition and sarcopenia in the elderly gastric cancer patients and investigate the predictive roles of malnutrition and sarcopenia on clinical outcomes.

## Methods and materials

### Patients

This study was a part of an observational study registered in the China Clinical Trial Registry (No. ChiCTR1800019717) and was approved by the Ethics Committee of The First Affiliated Hospital of Wenzhou Medical University. From August 2014 to September 2019, elderly patients (age ≥ 65 years old) with gastric cancer undergoing radical surgery in the department of gastrointestinal surgery from The First Affiliated Hospital of Wenzhou Medical University in Wenzhou were included in this study. Exclusion criteria were as following: (1) late stage of gastric cancer with metastasis; (2) palliative or emergency surgery; (3) patients have received oncological therapies before surgery such as chemotherapy or radiotherapy; (4) incomplete data. Standard radical gastrectomy with D2 lymphadenectomy for advanced stage or D1^+^ lymphadenectomy for early stage were performed based on guidelines (22, 23). All patients had signed informed consent forms before they participated in this study.

### Data collection

The following clinical data was prospectively collected and was deposited in the electronic medical database for analysis: age, gender, body mass index (BMI), preoperative albumin and hemoglobin concentration, American Society of Anesthesiologists (ASA) grade, Nutritional Risk Screening 2002 (NRS2002) score, Charlson Comorbidity Index (24), previous abdominal surgery, handgrip strength, third lumbar vertebra (L3) skeletal muscle index (SMI, cm^2^/m^2^), tumor location, differentiation of tumor, Tumor-Node-Metastasis (TNM) stage, type of gastrectomy, laparoscopic surgery, combined resection, surgical duration, postoperative complications, postoperative hospital stays, hospitalization costs, readmissions within 30 days of discharge, overall survival (OS) and disease-free survival (DFS). For the diagnosis of nutritional risk and malnutrition, a questionnaire survey about weight loss, reduced food intake, and general condition was conducted at the time of admission.

### Definition and grouping

Malnutrition was diagnosed based on the GLIM criteria (20). Because cancer is an etiologic component of the GLIM criteria, patients who met any phenotypic criterion in this study were considered with malnutrition. The cross-sectional computed tomography slice at the third lumbar vertebra (L3) was selected for muscle mass measurement. Sarcopenia was diagnosed according to revised consensus from the European Working Group on Sarcopenia in Older People (EWGSOP2) (7), the cutoff values of SMI were obtained from our previous study (25). Low handgrip strength was defined as < 18 kg for females and < 26 kg for males (26). Hypoalbuminemia was defined as serum albumin concentration < 35 g/L. Anemia was defined as hemoglobin concentration < 110 g/L for female and < 120 g/L for male. OS was defined as the time from surgery to death or last follow-up time. DFS was defined as the time from surgery to the time of recurrence or death for non-cancer cause.

Postoperative complications were graded by Clavien–Dindo classification (27). Complications with grade ≥ II were included for analysis. Complications with grade ≥ III were defined as severe complications. For classification of postoperative complications, surgical complications included gastrointestinal dysfunction (including delayed gastric emptying and prolonged postoperative ileus), wound infection, bleeding, seroperitoneum, anastomotic leakage, lymphorrhagia, pancreatic fistula and biliary fistula, and medical complications included pulmonary complication, cardiac complication, venous thrombosis, persistent hypoalbuminemia, cerebral infarction, sepsis, hepatic failure, and urinary complication.

Patients were divided into four groups according to presence of sarcopenia or malnutrition: normal group (NN), malnutrition-only group (MN), sarcopenia-only group (SC) and malnutrition-sarcopenia group (MS).

### Preoperative nutritional support

Nutritional risk assessment is contained in the routine preoperative management. NRS2002 tool was used to identify patients at high nutritional risk at the time of admission. Patients with NRS 2002 score ≥ 3 should receive a nutritional supplement through enteral or parenteral route for at least 1 week before surgery.

### Follow up

After discharge from hospital, all patients regularly participated in postoperative follow-up program by telephone interviews or outpatient visits. The follow-up program was conducted within the 1st month after surgery, every 3 months for the first 2 years, and every 6 months thereafter. The last follow-up date was February 2020.

### Statistical analysis

Continuous variables were expressed as mean and standard deviation (SD), or median and interquartile range (IQR). Categorical variables were expressed as counts and percentages. For comparison among four groups, Variance test or Kruskal-Wallis H test were used for continuous variables, and Pearson's Chi-square test or Fisher's exact test were used for categorical variables. Kaplan-Meier method and log-rank test were used for survival analysis. Univariate analysis and multivariate analyses (logistic regression or Cox proportional hazards regression) were used for independent risk factors for complications, OS and DFS. In the univariate analysis, variables with a *P* < 0.10 were included into subsequent multivariate analyses. Statistical significance was obtained when two-tailed *P* < 0.05. Statistical analyses were performed using the SPSS statistics version 22.0 (IBM Corp, IBM SPSS Statistics for Windows, Armonk, NY) and MedCalc Software version 15.2 (MedCalc, Ostend, Belgium).

## Results

### Patients' cohort

A total of 742 patients participated in this study (170 women, 572 men) with a median age of 72 (range: 65–91) years and a median BMI of 22.2 (range: 13.2–37.5) kg/m^2^. There were 250 (33.7%) patients with comorbidities. Median NRS2002 score was 2 and 333 (44.9%) patients had NRS2002 score ≥ 3. The median preoperative plasma albumin concentration of this study population was 37.1 (range: 20.9–49.9) g/L. Laparoscopic surgery was performed in 239 (32.2%) patients. Based on the diagnostic criteria, the prevalence of malnutrition and sarcopenia were 34.8% (*n* = 258) and 34.0% (*n* = 252), respectively. Of all patients, 17.5% (*n* = 130) had both two symptoms. The patients were classified into four groups: 362 patients in the NN group, 128 patients in the MN group, 122 patients in the SC group, and 130 patients in the MS group.

### Clinicopathologic characteristics

The demographic and clinicopathologic characteristics of each group were summarized in Table 1. Among these four groups, patients in the MS group had the oldest age (*P* < 0.001), lowest BMI (*P* < 0.001) lowest serum albumin (*P* < 0.001) and hemoglobin (*P* < 0.001), lowest L3-SMI (*P* < 0.001) and handgrip strength (*P* < 0.001), highest TNM stage (*P* < 0.001) and highest NRS2002 score (*P* < 0.001). In addition, there was the highest proportion of female in the MS group (*P* = 0.030).

**Table 1:** Comparison of demographic and clinical characteristic of patients.

**Factors**	**Total (*n =* 742)**	**NN (*n =* 362)**	**MN (*n =* 128)**	**SC (*n =* 122)**	**MS (*n =* 130)**	***P***
Age, median (IQR), years	72 (8)	70 (7)	71.5 (7)	74 (9)	76 (8)	<0.001^*^
Gender						0.030^*^
Male	572 (77.1)	290 (80.1)	93 (72.7)	99 (81.1)	90 (69.2)	
Female	170 (22.9)	72 (19.9)	35 (27.3)	23 (18.9)	40 (30.8)	
BMI, median (IQR), kg/m^2^	22.2 (4.0)	23.4 (3.6)	20.3 (4.0)	22.8 (3.4)	19.6 (3.3)	<0.001^*^
Albumin, median (IQR), g/L	37.1 (5.8)	38.6 (5.1)	36.2 (5.1)	37.0 (5.5)	34.1 (6.5)	<0.001^*^
Hemoglobin, median (IQR), g/L	120 (34)	126.5 (29)	115.5 (31)	116 (38.5)	108 (31.3)	<0.001^*^
L3-SMI, mean (SD), cm^2^/m^2^	41.5 (7.5)	44.6 (6.8)	38.8 (6.6)	41.7 (6.4)	35.1 (5.7)	<0.001^*^
Handgrip strength, median (IQR), kg	24.9 (12.5)	29.0 (9.4)	27.9 (11.0)	19.3 (7.5)	16.7 (9.3)	<0.001^*^
ASA grade						0.114
I	249 (33.6)	120 (33.1)	40 (31.3)	48 (39.3)	41 (31.5)	
II	379 (51.1)	199 (55.0)	62 (48.4)	53 (43.4)	65 (50.0)	
III	114 (15.4)	43 (11.9)	26 (20.3)	21 (17.2)	24 (18.5)	
NRS 2002 scores, median (IQR)	2 (2)	2 (1)	4 (2)	2 (0)	4 (2)	<0.001^*^
Charlson comorbidity index						0.341
0	492 (66.3)	244 (67.4)	91 (71.1)	72 (59.0)	85 (65.4)	
1	168 (22.6)	81 (22.4)	26 (20.3)	35 (28.7)	26 (20.0)	
≥ 2	82 (11.1)	37 (10.2)	11 (8.6)	15 (12.3)	19 (14.6)	
Previous abdominal surgery	93 (12.5)	42 (11.6)	15 (11.7)	19 (15.6)	17 (13.1)	0.699
Tumor location						0.286
Proximal	128 (17.3)	72 (19.9)	17 (13.3)	20 (16.4)	19 (14.6)	
Medium	161 (21.7)	86 (23.8)	27 (21.1)	25 (20.5)	23 (17.7)	
Distal	430 (58.0)	196 (54.1)	78 (60.9)	71 (58.2)	85 (65.4)	
2/3 or more	23 (3.1)	8 (2.2)	6 (4.7)	6 (4.9)	3 (2.3)	
Differentiation of tumor						0.287
Well-differentiated	271 (36.5)	144 (39.8)	41 (32.0)	44 (36.1)	42 (32.3)	
Poorly differentiated	471 (63.5)	218 (60.2)	87 (68.0)	78 (63.9)	88 (67.7)	
TNM stage						<0.001^*^
I	255 (34.4)	158 (43.6)	32 (25.0)	34 (27.9)	31 (23.8)	
II	197 (26.5)	95 (26.2)	34 (26.6)	32 (26.2)	36 (27.7)	
III	290 (39.1)	109 (30.1)	62 (48.4)	56 (45.9)	63 (48.5)	
Type of gastrectomy						0.453
Subtotal gastrectomy	448 (60.4)	211 (58.3)	83 (64.8)	71 (58.2)	83 (63.8)	
Total gastrectomy	294 (39.6)	151 (41.7)	45 (35.2)	51 (41.8)	47 (36.2)	
Combined resection	58 (7.8)	23 (6.4)	10 (7.8)	10 (8.2)	15 (11.5)	0.308
Laparoscopic surgery	239 (32.2)	132 (36.5)	37 (28.9)	37 (30.3)	33 (25.4)	0.085

Values in parentheses are percentages unless indicated otherwise.

* Statistically significant (P < 0.05).

NN, Normal group; MN, Malnutrition-only group; SC, Sarcopenia-only group; MS, Malnutrition - sarcopenia group; SD, Standard Deviation; IQR, Interquartile Range; BMI, Body Mass Index; ASA, American Society of Anesthesiologists; L3-SMI, L3 skeletal muscle index NRS, nutritional risk screening; TNM, tumor–node–metastasis.

### Short-term outcomes

Table 2 showed the comparison of postoperative outcomes of each group. The incidence of total postoperative complications in the entire cohort was 25.5%. Patients in the MS group had the highest rate of total complication (18.8 vs. 27.7 vs. 32.0 vs. 40.8%, respectively, *P* < 0.001), surgical complications (11.3 vs. 15.6 vs. 18.9 vs. 24.6%, respectively, *P* = 0.003), and medical complications (9.7 vs. 10.2 vs. 14.8 vs. 19.2%, respectively, *P* = 0.025). They also had the highest postoperative hospital stays (*P* < 0.001) and hospitalization costs (*P* < 0.001). Moreover, the four groups did not show a significant difference in the rate of severe complications, surgical durations, and readmission rate. Univariate and multivariate analyses for risk factors for total complications were presented in Table 3. In the present study, sarcopenia [Odds ratio 2.279 (1.620–3.206); *P* < 0.001] and Charlson Comorbidity Index [≥2, Odds ratio 1.924 (1.163–3.181); *P* = 0.011] were independent risk factors for total complications.

**Table 2:** Postoperative outcomes of each group.

**Factors**	**Total**	**NN**	**MN**	**SC**	**MS**	***P***
	**(*n =* 742)**	**(*n =* 362)**	**(*n =* 128)**	**(*n =* 122)**	**(*n =* 130)**	
Total complications	189 (25.5)	68 (18.8)	29 (22.7)	39 (32.0)	53 (40.8)	<0.001^*^
Surgical complications	116 (15.6)	41 (11.3)	20 (15.6)	23 (18.9)	32 (24.6)	0.003^*^
Medical complications	91 (12.3)	35 (9.7)	13 (10.2)	18 (14.8)	25 (19.2)	0.025^*^
Severe complications	56 (7.5)	21 (5.8)	9 (7.0)	11 (9.0)	15 (11.5)	0.174
Surgical durations, median (IQR), minutes	200 (75)	200 (70)	202.5 (75)	202.5 (70)	190 (90)	0.486
Postoperative hospital stays, median (IQR), days	14 (8)	13 (6)	13 (8)	14 (9)	16.5 (12)	<0.001^*^
Costs, median (IQR), yuan	63715.1 (24630.0)	59996.4 (21301.9)	62328.8 (27616.4)	67934.5 (24399.5)	74193.3 (32180.6)	<0.001^*^
Readmissions within 30 days of discharge	43 (5.8)	17 (4.7)	5 (3.9)	9 (7.4)	12 (9.2)	0.171

Values in parentheses are percentages unless indicated otherwise.

* Statistically significant (P < 0.05).

NN, Normal group; MN, Malnutrition-only group; SC, Sarcopenia-only group; MS, Malnutrition - sarcopenia group; IQR, Interquartile Range.

**Table 3:** Univariate and multivariate analysis for risk factors of total complications.

**Factors**	**Univariate analysis**		**Multivariate analysis**	
	**OR (95% CI)**	***P***	**OR (95% CI)**	***P***
Gender
Male/female	0.918 (0.609–1.384)	0.682		
BMI
≤18.5/18.5–25	0.907 (0.486–1.693)	0.760		
>25/18.5–25	0.985 (0.619–1.569)	0.949		
Hypoalbuminemia
Yes/no	1.314 (0.872–1.978)	0.191		
Anemia
Yes/no	0.967 (0.646–1.449)	0.872		
GLIM-defined malnutrition				
Yes/no	1.322 (0.885–1.975)	0.173		
Sarcopenia
Yes/no	1.982 (1.378–2.852)	<0.001^*^	2.279 (1.620–3.206)	<0.001^*^
TNM stage
III/I	1.073 (0.674–1.709)	0.766		
II/I	1.215 (0.758–1.950)	0.419		
Charlson comorbidity index
1/0	1.228 (0.812–1.859)	0.331		
≥2/0	1.869 (1.114–3.137)	0.018^*^	1.924 (1.163–3.181)	0.011^*^
Previous abdominal surgery
Yes/no	1.409 (0.863–2.301)	0.171		
Differentiation of tumor
Poorly/well	1.127 (0.765–1.660)	0.544		
Type of resection
Total/Subtotal	1.105 (0.772–1.580)	0.586		
Combined resection				
Yes/no	1.184 (0.642–2.181)	0.588		
Laparoscopic surgery
Yes/no	0.924 (0.627–1.362)	0.690		

* Statistically significant (P < 0.05).

OR, Odds Ratio; CI, Confidence Interval; BMI, Body mass index; ASA, American Society of Anesthesiologists; TNM, tumor–node–metastasis.

### Survival

**Figure 1:**
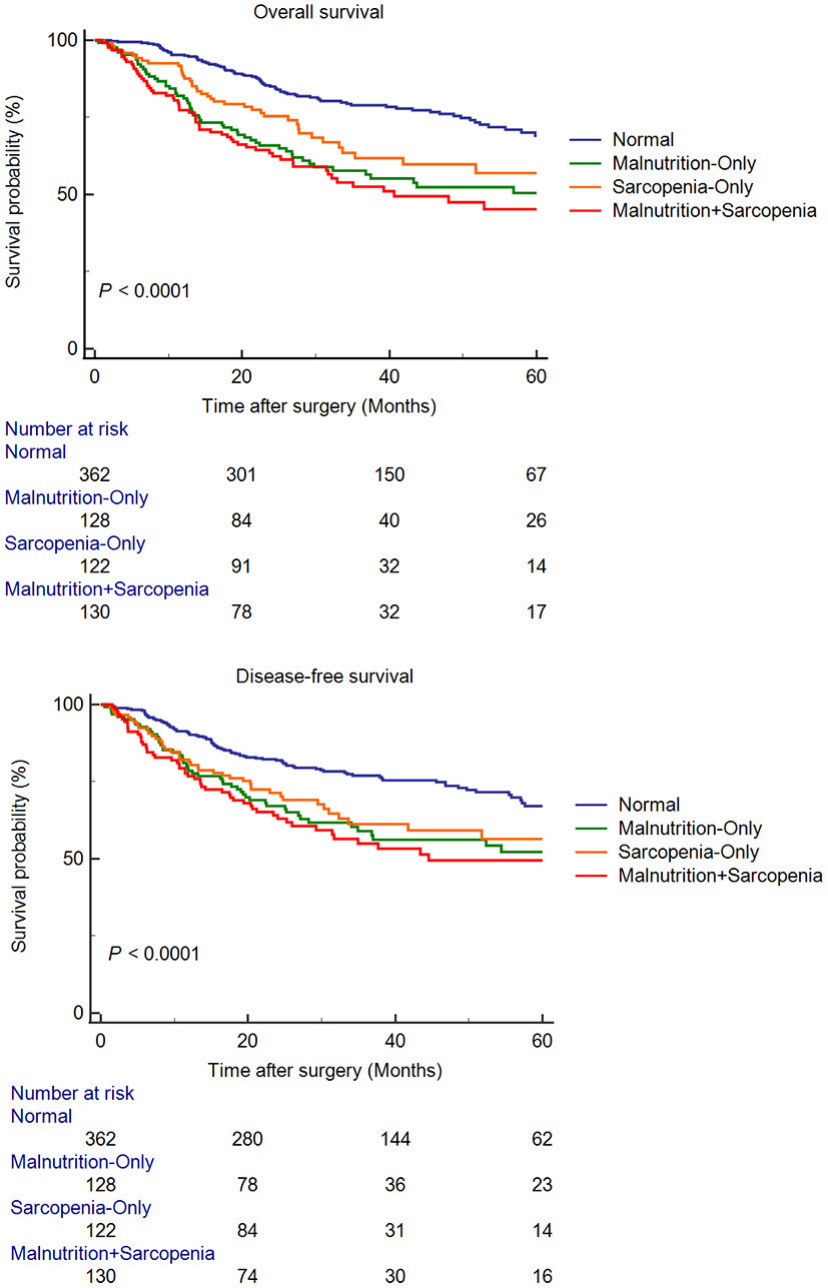
Kaplan-Meier curves for overall survival and disease-free survival, according to presence of malnutrition and sarcopenia.

The median follow up time was 32.35 months. For the entire cohort, the 1-, 3- and 5-year OS rates were 88.7, 68.1, and 59.9%, respectively; the 1-, 3- and 5-year DFS rates were 84.8, 67.6, and 59.6%, respectively. Figure 1 showed the survival curves for OS and DFS of four groups. The comparation indicated that among four groups, patients in the MS group had the worst OS (*P* < 0.0001) and DFS (*P* < 0.0001). The independent risk factors for OS and DFS were shown in Tables 4, 5, respectively. hypoalbuminemia [Hazard Ratio 1.408 (1.085–1.827); *P* = 0.010], GLIM-defined malnutrition [Hazard Ratio 1.742 (1.345–2.256); *P* < 0.001], and TNM stage [III: Hazard Ratio 6.707 (4.411–10.199); *P* < 0.001, II: Hazard Ratio 2.560 (1.602–4.093); *P* < 0.001] were independently associated with poor OS. In terms of DFS, hypoalbuminemia [Hazard Ratio 1.497 (1.152–1.945); *P* = 0.003], GLIM-defined malnutrition [Hazard Ratio 1.359 (1.045–1.767); *P* = 0.022], TNM stage [III: Hazard Ratio 7.873 (4.960–12.499); *P* < 0.001, II: Hazard Ratio 2.741 (1.650–4.553); *P* < 0.001], and poor differentiation of tumor [Hazard Ratio 1.456 (1.057–2.005); *P* = 0.021] were identified as independent risk factors.

**Table 4:** Univariate and multivariate analysis for risk factors of overall survival.

**Factors**	**Univariate analysis**		**Multivariate analysis**	
	**OR (95% CI)**	***P***	**OR (95% CI)**	***P***
Gender
Male/female	1.051 (0.762–1.450)	0.761		
BMI
≤18.5/18.5–25	0.780 (0.495–1.228)	0.283		
>25/18.5–25	0.883 (0.615–1.268)	0.501		
Hypoalbuminemia
Yes/no	1.297 (0.966–1.743)	0.084	1.408 (1.085–1.827)	0.010^*^
Anemia
Yes/no	1.183 (0.872–1.603)	0.280		
GLIM-defined malnutrition
Yes/no	1.776 (1.338–2.356)	<0.001^*^	1.742 (1.345–2.256)	<0.001^*^
Sarcopenia
Yes/no	1.080 (0.821–1.421)	0.583		
TNM stage
III/I	5.410 (3.449–8.485)	<0.001^*^	6.707 (4.411–10.199)	<0.001^*^
II/I	2.174 (1.334–3.545)	0.002^*^	2.560 (1.602–4.093)	<0.001^*^
Charlson comorbidity index
1/0	1.118 (0.815–1.533)	0.488		
≥2/0	1.028 (0682–1.550)	0.896		
Previous abdominal surgery
Yes/no	0.764 (0.497–1.174)	0.219		
Differentiation of tumor
Poorly/well	1.359 (0.992–1.862)	0.056		
Type of resection
Total/Subtotal	1.023 (0.784–1.334)	0.869		
Combined resection
Yes/no	1.212 (0.795–1.848)	0.372		
Laparoscopic surgery
Yes/no	0.826 (0.595–1.147)	0.254		

* Statistically significant (P < 0.05).

HR, Hazard Ratio; CI, Confidence Interval; BMI, Body mass index; ASA, American Society of Anesthesiologists; TNM, tumor–node–metastasis.

**Table 5:** Univariate and multivariate analysis for risk factors of disease-free survival.

**Factors**	**Univariate analysis**		**Multivariate analysis**	
	**OR (95% CI)**	***P***	**OR (95% CI)**	***P***
Gender
Male/female	1.156 (0.829–1.610)	0.393		
BMI
≤18.5/18.5–25	0.884 (0.548–1.425)	0.612		
>25/18.5–25	0.817 (0.569–1.172)	0.271		
Hypoalbuminemia
Yes/no	1.442 (1.072–1.941)	0.016^*^	1.497 (1.152–1.945)	0.003^*^
Anemia
Yes/no	1.029 (0.760–1.394)	0.854		
GLIM-defined malnutrition
Yes/no	1.372 (1.027–1.833)	0.032^*^	1.359 (1.045–1.767)	0.022^*^
Sarcopenia
Yes/no	1.026 (0.777–1.355)	0.857		
TNM stage
III/I	7.301 (4.530–11.765)	<0.001^*^	7.873 (4.960–12.499)	<0.001^*^
II/I	2.615 (1.557–4.392)	<0.001^*^	2.741 (1.650–4.553)	<0.001^*^
Charlson comorbidity index
1/0	1.221 (0.890–1.675)	0.217		
≥2/0	1.085 (0.722–1.629)	0.695		
Previous abdominal surgery
Yes/no	0.859 (0.563–1.311)	0.481		
Differentiation of tumor
Poorly/well	1.476 (1.066–2.044)	0.019^*^	1.456 (1.057–2.005)	0.021^*^
Type of resection
Total/Subtotal	0.996 (0.763–1.301)	0.978		
Combined resection
Yes/no	1.390 (0.912–2.118)	0.126		
Laparoscopic surgery
Yes/no	0.783 (0.562–1.091)	0.149		

* Statistically significant (P < 0.05).

HR, Hazard Ratio; CI, Confidence Interval; BMI, Body mass index; ASA, American Society of Anesthesiologists; TNM, tumor–node–metastasis.

## Discussion

Low dietary intake, increased catabolism of protein and fat, and increased inflammatory factors due to aging and malignancy have made elderly gastric cancer patients more susceptible to sarcopenia and malnutrition. This is becoming an increasingly important issue in a rapidly aging society. Although the clinical manifestations of the two are similar in real world, the underlying mechanisms of the onset and development are considered different. Previous studies have examined only the effect of either symptom on elderly gastric cancer patients. To date, no reports on the effects of the coexistence of these two symptoms on elderly gastric cancer patients after surgery are available. This study strictly followed the EWGSOP2 and GLIM diagnostic criteria in the diagnosis of sarcopenia and malnutrition, respectively. Further, screening for the nutritional risks was performed before the diagnosis of malnutrition. This study included 742 elderly gastric cancer patients, among whom the incidence of sarcopenia, malnutrition, and both conditions were 34.0, 34.8, and 17.5%, respectively. Due to the high prevalence and the overlap in the diagnostic criteria, it is reasonable that elderly gastric cancer patients suffer from both symptoms. This demonstrated the possibility of the coexistence of sarcopenia and malnutrition. Moreover, similar incidence rates were observed for both symptoms in the elderly gastric cancer patients. Since the diagnostic criteria for both diseases included the assessment of muscle mass, the importance of muscle-related assessment in evaluating the functional capacity of elderly patients was highlighted. However, the GLIM criteria included items not included in the diagnosis of sarcopenia. It seems that sarcopenia mainly represents the status of the muscles while malnutrition represents the status of the whole body. These are the differences between these two symptoms. In addition, the elderly patients with malnutrition as diagnosed according to the GLIM criteria have been reported to present a significantly increased risk of sarcopenia (28), and that malnutrition may play an important role in the sarcopenia management (29). Furthermore, malnutrition may lead to sarcopenia due to the insufficient intake of protein that is necessary to maintain muscle mass. In turn, sarcopenia may exacerbate malnutrition by reducing dietary intake and daily activity (30). Therefore, evaluating both sarcopenia and malnutrition was essential in the preoperative assessment of elderly gastric cancer patients. Moreover, the early identification of individuals at risk for malnutrition may be the key to preventing sarcopenia and reducing their health burdens, which can help identify the population at high surgical risk preoperatively.

By comparing the clinical characteristics in each group, the elderly gastric cancer patients with both sarcopenia and malnutrition were found to present the oldest age, lowest BMI, lowest albumin and hemoglobin levels, lowest muscle mass, and poorest muscle function. In addition, the highest proportion of TNM stage III patients among all groups was observed in this group. Moreover, these patients had the worst preoperative functional status compared to that of the three other groups, suggesting that sarcopenia and malnutrition had synergistic effects on elderly gastric cancer patients and jointly reduced body reserves and functions. Elderly patients have difficulties overcoming major surgeries due to the multiple adverse conditions of age, tumor, malnutrition, and sarcopenia, thereby presenting a great challenge to surgeons, and requiring careful surgical strategy.

Regarding the short-term postoperative outcomes among all groups, elderly gastric cancer patients with both sarcopenia and malnutrition had the highest rates of total, surgical, and medical complications, longest postoperative hospital stay, and highest hospitalization costs, confirming the superimposed negative effects of malnutrition and sarcopenia. Sarcopenia and malnutrition are both associated with a negative protein balance caused by reduced food intake and enhanced catabolism of cancer, subsequently expose elderly patients to negative clinical outcomes such as higher risk of postoperative complications and poorer quality of life. In addition, sarcopenia was demonstrated to be an independent risk factor for postoperative complications; hence, it might impact the short-term postoperative outcomes more than those due to malnutrition. Sarcopenia may lead to decreased respiratory-related muscle function, which can cause more severe dyspnea, poorer lung function, and lower exercise test results (31). Since general anesthesia is required for large procedures, such as radical gastrectomy, elderly gastric cancer patients with combined malnutrition and sarcopenia have difficulty in quick recovery from such procedures. In addition, sarcopenia can decrease the motor capacity in elderly patients, which limits the postoperative movements and reduces out-of-bed activities in patients, thereby preventing postoperative recovery, extending hospital stays, increasing hospitalization expenses, and thus posing great financial pressure on patients' families and reducing the effectiveness of surgeries.

Survival analysis suggested that among these four groups, elderly gastric cancer patients with both malnutrition and sarcopenia had the worst OS and DFS. Apparently, the coexistence of both might increase the risk of death and recurrence in elderly gastric cancer patients. In addition, it is important to note that these patients with both symptoms are excessively fragile and have poorer tolerance to postoperative chemotherapy, experience more chemotherapy-related toxicity and poorer survival. Multivariate analysis showed that hypoproteinemia and malnutrition were non-tumor-related independent risk factors for OS and DFS, while sarcopenia was not. This suggested that malnutrition impacted the long-term outcomes more than those due to sarcopenia. In contrast, it demonstrated that sarcopenia could not be used to fully describe the nutritional status of elderly gastric cancer patients. Malnutrition has been reported to be associated with mortality in elderly communities aged > 85 years (32). In addition, malnutrition, diagnosed according to the GLIM criteria, is associated with a 4.4-fold increase in the death risk in the elderly population with sarcopenia (33). Moreover, malnutrition is associated with all-cause mortality regardless of the tumor type and other risk factors (34). Malnutrition may lead to a postoperative decline in physical functions, severely affect the quality of life, and impede subsequent anticancer therapies in elderly gastric cancer patients. In addition, malnutrition commonly causes secondary immune dysfunction. Patients with poor nutritional status present poor OS after immunotherapy; this may increase the risk of tumor recurrence after radical gastrectomy (35). Therefore, poor nutritional status may be a reason for the poor long-term prognosis in elderly gastric cancer patients.

The strength of this study was that this was the first large-scale study to describe how the coexistence of malnutrition and sarcopenia affected the prognosis of elderly gastric cancer patients. However, this study has some potential limitations. First, this was a single-center study. Future multicenter studies are needed to validate the results due to the differences in ethnicity or physical fitness between the Eastern and Western populations. Furthermore, specific simultaneous screening for these two symptoms was lacking. Future research should focus on developing simple and easy-to-apply screening tools including muscle assessment for MSS. In addition, preoperative hypoproteinemia was found to be an independent risk factor for long-term prognosis in this study, and serum albumin levels might be used as a biochemical indicator in the screening tool. Last, muscle mass is widely accepted to be restored through nutritional management and preoperative rehabilitation (36, 37). Exercise has been shown to increase muscle mass and function and reduce systemic inflammation, thereby reducing the symptoms of cachexia and sarcopenia (38). Therefore, early therapeutic interventions, including nutritional support, may have positive impacts on the prognosis of elderly gastric cancer patients. However, the inability to include these in their daily physical activity program by some elderly people should be considered. In addition, the elderly population is heterogeneous, and the MSS treatment should be maximally individualized. The effect of postoperative nutritional status on elderly gastric cancer patients is also unknown. These questions should also be addressed in future studies.

## Conclusions

In conclusion, elderly gastric cancer patients with both malnutrition and sarcopenia presented the highest rate of postoperative complications and worst OS and DFS. Sarcopenia and malnutrition affected the short- and long-term outcomes, respectively, thereby exerting simultaneous superimposed negative effects. This study recommended screening for malnutrition and sarcopenia in all elderly gastric cancer patients, regardless of the severity, during the preoperative geriatric evaluation to provide useful information for risk categorization and selection of the optimal treatment strategy.

## Data availability statement

The original contributions presented in the study are included in the article/supplementary materials, further inquiries can be directed to the corresponding author/s.

## Ethics statement

The studies involving human participants were reviewed and approved by the Ethics Committee of the First Affiliated Hospital of Wenzhou Medical University. The patients/participants provided their written informed consent to participate in this study.

## Author contributions

C-LZ and ZY conceived and designed the study and reviewed the paper and made the decision to submit the article for publication. W-ZC, W-HC, and Q-TD collected the data. D-YY, X-ZZ, FL, and F-MZ analyzed and interpreted the data. X-ZZ and W-ZC wrote and revised the paper. All authors read and approved the final manuscript.

## Funding

This study was supported by National Natural Science Foundation of China (No. 82171565), Science and Technology Commission of Shanghai Municipality (No. 16411954200), Shanghai Association of Integrative Medicine (No. shcim202101), and Shanghai Municipal Health and Family Planning Commission (No. 21DZ2208300).

## Conflict of interest

The authors declare that the research was conducted in the absence of any commercial or financial relationships that could be construed as a potential conflict of interest.

## Publisher's note

All claims expressed in this article are solely those of the authors and do not necessarily represent those of their affiliated organizations, or those of the publisher, the editors and the reviewers. Any product that may be evaluated in this article, or claim that may be made by its manufacturer, is not guaranteed or endorsed by the publisher.
